# The large GTPase Rab44 regulates granule exocytosis in mast cells and IgE-mediated anaphylaxis

**DOI:** 10.1038/s41423-020-0413-z

**Published:** 2020-04-01

**Authors:** Tomoko Kadowaki, Yu Yamaguchi, Mizuho A. Kido, Takaya Abe, Kohei Ogawa, Mitsuko Tokuhisa, Weiqi Gao, Kuniaki Okamoto, Hiroshi Kiyonari, Takayuki Tsukuba

**Affiliations:** 1 Department of Frontier Life Science, Graduate School of Biomedical Sciences, Nagasaki University, Nagasaki, 8528588 Japan; 2 Department of Dental Pharmacology, Graduate School of Biomedical Sciences, Nagasaki University, Nagasaki, 8528588 Japan; 3 Department of Anatomy and Physiology, Faculty of Medicine, Saga University, Saga, 849-8501 Japan; 4 Laboratory for Animal Resources and Genetic Engineering, RIKEN Center for Biosystems Dynamics Research, 2-2-3 Minatojima Minami-machi, Chuou-ku, Kobe, 6500047 Japan; 5 Department of Dental Pharmacology, Graduate School of Medicine, Dentistry and Pharmaceutical Sciences, Okayama University, Okayama, 7008525 Japan

**Keywords:** Mast cells, Secretion

Mast cells are responsible for anaphylaxis and allergy and regulate various innate and adaptive immune responses.^1,2^ Although several “small” Rab GTPases were reported to regulate exocytosis of mast cells,^3–5^ little is known about the contribution of “large” Rab GTPases. Rab44 is a large Rab-GTPase that contains a Rab-GTPase domain and some additional N-terminal domains.^6,7^ Here, we investigated the role of Rab44 in the physiology of mast cells and in anaphylaxis. Rab44 was expressed as two isoforms in murine bone-marrow mast cells (BMMCs), both of which were deleted in *Rab44*-knockout mice. The *Rab44*-knockout mice exhibited diminished anaphylaxis, and the *Rab44*-knockout BMMCs showed a decrease in FcεRI-mediated histamine and β-hexosaminidase secretion. Thus, Rab44 regulates granule exocytosis in mast cells and IgE-mediated anaphylaxis in mice.

By analyzing the systemic distribution of Rab44 in mice, we found that Rab44 was highly expressed in bone marrow. Immunohistochemical analysis of bone marrow indicated that Rab44 immunoreactivity was observed in granulocyte- or mast cell-lineage cells filled with granules with few actin filaments (Fig. 1a, b, enlarged view). Quantitative RT-PCR analysis indicated that the *Rab44* mRNA expression levels were 8.4-times higher in the bone marrow-derived mast cells (BMMCs) than in the bone marrow (Fig. 1c).

**Fig. 1:**
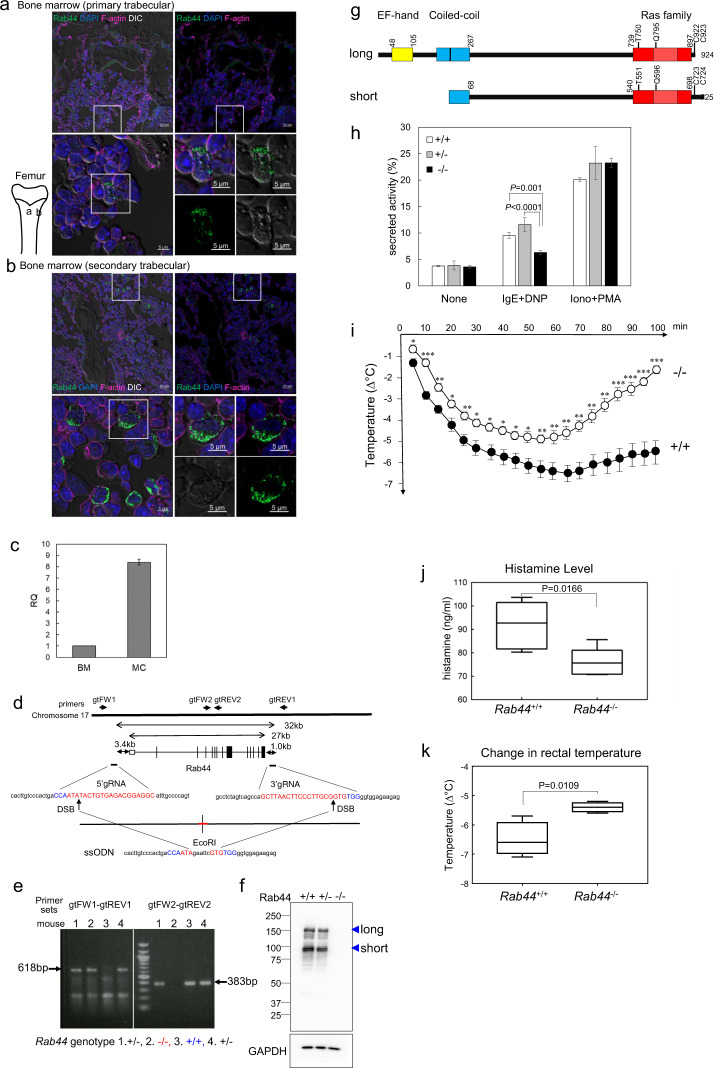
**a** Immunofluorescence confocal images of Rab44 in the primary trabecular bone of the mouse femur. **b** Immunofluorescence confocal images of Rab44 in the mouse bone marrow of secondary trabecular bone. Note that the Rab44-immunoreactive cells were filled with abundant granules. **c** Quantitative RT-PCR of Rab44 in the bone marrow and the BMMCs. **d** Schematic illustration of the *Rab44* gene structure and targeting scheme. The gRNA targets and protospacer adjacent motives (PAMs) are indicated in red and blue, respectively. Predicted Cas9 cutting sites are shown with arrows. Knock in of an EcoRI digestion site within the deletion site by ssODN. **e** Genotyping PCR indicated an extra 618-bp band as a result of gene editing. A 383-bp band disappeared, as shown by PCR using primers that hybridized to the internal sites of the *Rab44* gene. **f** The expression level of the Rab44 protein in the BMMCs was examined by western blotting. **g** Transcripts of mouse *Rab44*. Rab44 was expressed as two isoforms by alternative splicing in the mouse BMMCs. **h** β-hexosaminidase secretion from the BMMCs derived from the *Rab44*^*+/+*^, *Rab44*^*+/*−^ and *RAb44*^−/−^ mice after sensitization with anti-DNP IgE and stimulation with DNP or ionomycin and PMA. **i** Rectal temperatures of the *Rab44*^*+/+*^ and *Rab44*^−/−^ mice after sensitization with anti-DNP IgE and challenge with DNP-HSA. Values represent the change from the prechallenge temperatures. **j** Plasma histamine levels of the mice subjected to passive anaphylaxis. **k** Rectal temperatures of the *Rab44*^*+/+*^ and *Rab44*^−/−^ mice sensitized and challenged with OVA. Data: In (**h**), values represent the ratio of secreted activity to total activity. In (**i**, **j**), values represent the change from the prechallenge temperatures *P < 0.05; **P < 0.01; ***P < 0.0001. Statistical tests: Tukey’s multiple comparison test. **h**–**k** Two-tailed unpaired *t* test

We generated Rab44-knockout mice using CRISPR/Cas9-mediated genomic editing. The guide sequences targeting the 5′ and 3′ regions of the *Rab44* gene were designed to delete a 32-kb genomic region spanning 13 exons of *Rab44* on chromosome 17 (Fig. 1d). A single-stranded oligonucleotide was designed to insert an *Eco*RI digestion site within the deleted region to confirm successful gene editing. The mice were confirmed as heterozygous for the gene deletion (*Rab44*^+/−^) and showed no apparent phenotypes (Fig. 1e). After we crossed the *Rab44*^+/−^ mice, homozygous (*Rab44*^−/−^) mice were born at the expected Mendelian frequency and showed no abnormal appearances.

Western blotting analysis of BMMC proteins was used to confirm the Rab44 deficiency at the protein level. Rab44 was shown to be expressed as two isoforms, termed the “long form”, with a molecular mass of approximately 160 kDa, and the “short form” of ~95 kDa (Fig. 1f). Although our previous study indicated that the short form was extensively expressed in osteoclasts,^7^ we first reported that the mouse Rab44 protein contained an EF-hand domain at the N-terminus in this study. The BMMCs from the *Rab44*^−/−^ mice were shown to be deficient in both isoforms of Rab44 (Fig. 1f, g). Sequence analysis of cDNAs was used to compare the gene structure of the long and short forms of mouse Rab44 with human Rab44. The long form of mouse Rab44 consisted of EF hand, coiled-coil, and Rab domains, similar to human Rab44; however, 47 amino acids in the coiled-coil domain and 38 amino acids in the region between coiled-coil and Rab domains were deleted in the mouse long form (Fig. 1g). The short form of mouse Rab44 had deletions of the EF hand and the N-terminal half of the coiled-coil domain.

To determine the effects of Rab44 on the exocytosis of mast cells in vitro, we sensitized the BMMCs from the wild-type (*Rab44*^+/+^), *Rab44*^+/−^, and *Rab44*^−/−^ mice with an anti-DNP IgE antibody. Upon stimulation with DNP-human serum albumin (HSA), the *Rab44*^−/−^ BMMCs showed significantly reduced β-hexosaminidase secretion compared with the *Rab44*^+/+^ and *Rab44*^+/−^ BMMCs (Fig. 1h). Upon stimulation with ionomycin/phorbol 12-myristate 13-acetate (PMA), which triggers Ca^2+^ influx and PKC activation,^3^ the total amount of β-hexosaminidase secretion was comparable among the BMMCs of the three genotypes (*Rab44*^+/+^, *Rab44*^+/−^, and *Rab44*^−/−^). These results indicate that Rab44 deficiency reduces FcεRI-mediated, but not ionomycin/PMA-stimulated, β-hexosaminidase secretion by BMMCs.

To compare passive systemic anaphylaxis in vivo, we sensitized the *Rab44*^+/+^ and *Rab44*^−/−^ mice with anti-DNP IgE and challenged them with DNP-HSA. *Rab44*^+/+^ mice displayed a marked reduction in rectal temperature to ~6 °C lower than the basal temperature (Fig. 1i). However, the *Rab44*^*−/−*^ mice showed a significantly lower level of rectal temperature fluctuation than the *Rab44*^+/+^ mice. Under these conditions, the histamine levels in the plasma of the *Rab44*^*−/−*^ mice were significantly lower than those in the *Rab44*^+/+^ mice (Fig. 1j). When we further investigated active systemic anaphylaxis with ovalbumin, we found that the *Rab44*^*−/−*^ mice showed a significant reduction in rectal temperature fluctuation compared with the *Rab44*^+/+^ mice (Fig. 1k).

In this study, we demonstrated that Rab44 was expressed as two isoforms by alternative splicing in the mouse BMMCs. When the *Rab44*^−/−^ mice were generated by CRISPR/Cas9-mediated genomic editing, they showed reduced anaphylactic responses in vivo. Moreover, the Rab44-deficient BMMCs showed reduced FcεRI-mediated β-hexosaminidase secretion in vitro.

## Supplementary information

Supplementary Materials and Methods

Supplementary Table
