# Supplementary material for: The large GTPase Rab44 regulates granule exocytosis in mast cells and IgE-mediated anaphylaxis
Source: Cell Mol Immunol. 2020 Apr 1;17(12):1287–9. doi: 10.1038/s41423-020-0413-z (PMC7784977; doi:10.1038/s41423-020-0413-z)
Supplement: Supplementary file 1 — Supplementary Materials and Methods [file 41423_2020_413_MOESM1_ESM.docx]

**Supplementary Materials and Methods**

**Ethics statement**

All protocols were approved by Institutional Animal Care and Use Committee (protocol 170220-1-4/1703071365-4) of Nagasaki University and the Institutional Animal Care and Use Committee (IACUC) of RIKEN Kobe Branch.

**Antibodies and reagents**

Mouse monoclonal anti- GAPDH (M171-3) antibody was purchased from Medical & Biological Laboratories. Monoclonal anti-DNP IgE was from Sigma-Aldrich. Antibodies against Rab44 were raised in rabbits using a recombinant protein, prepared as described below. The full-length sequence (2175 bp) of the mouse short-type *Rab44* was PCR-amplified from the cDNA derived from bone marrow cells. The *Nde*I–*Xho*I fragment, including *Rab44*, was inserted into the expression vector, pET28a (+) (Merk Novagen, Darmstadt, Germany). The pET28a (+) plasmids containing N-terminal His_6_-tagged Rab44 were transformed into *E. coli* BL21(DE3) and gene expression was induced by the addition of isopropyl-β-D-thiogalactopyranoside. The recombinant Rab44 was purified using nickel–nitrilotriacetic acid agarose (Thermo Fisher Scientific). Rabbit antiserum was subjected to purification by column chromatography, using protein G-sepharose, followed by recombinant Rab44-conjugated agarose beads, to obtain the specific IgG against Rab44. *p*-nitrophenyl-N-acetyl-β-D-glucosaminide (N9376) and 2,4- DNP-HSA (A6661) were from Sigma-Aldrich. The histamine ELISA kit was from Enzo Life Sciences. Ionomycin and PMA were from FUJIFILM-WAKO.

**Cell culture**

Bone marrow cells from 8-week-old mice were cultured at 37°C and 5% CO_2_ in RPMI 1640 medium containing 10% fetal bovine serum, 2 mM L-glutamine, 5 × 10^-5^ M 2-mercaptoethanol, 1% penicillin and streptomycin, and 30% WEHI-3-conditioned medium as a source of IL-3. After 5 d, non-adherent cells were cultured at 10^5^ cells/mL in fresh complete medium containing WEHI-3-conditioned medium. After 4 weeks of culture, with the medium exchanged for fresh complete medium every third day, greater than 95% of cells were confirmed as BMMCs by toluidine blue staining.

**Quantitative RT-PCR.**

Total RNA was transcribed into cDNA using ReverTra Ace qPCR RT master mix (Toyobo). Quantitative RT-PCR was performed using the Brilliant III UltraFast SYBR Green QPCR Master Mix (Agilent Technologies) and an Applied Biosystems StepOne Plus real-time PCR system (Thermo Fisher Scientific). Primers for quantitative PCR (supplementary Table S1) were designed using Primer3 software (http://primer3.sourceforge.net/). The expression level of each targeted gene was normalized to *GAPDH* expression. All PCRs were performed in triplicate. The efficiency of primer binding was determined using linear regression by plotting the cycle threshold (*C_T_*) value versus the log of the cDNA dilution. Experiments were performed on three independent occasions, with comparable results.

**Generation of Rab44 knockout mice**

Rab44-knockout mice were selected in a genetic background of C57BL/6. We generated mice lacking the *Rab44* gene using CRISPR/Cas9-mediated genomic editing. As shown in Fig2A, the guide sequences targeting the 5′ and 3′ region of the mouse *Rab44* gene were designed to delete large genomic regions spanning 13 exons. Knock-in of an EcoRI digestion site within the deleted region was mediated by ssODN. The gRNA and Cas9 protein were introduced into the mouse zygotes by electroporation^8^. To confirm the insertion product, we performed PCR of the tail DNA from F0 mice, and DNA sequencing. Four mice were confirmed to have the mutationfrom a total of 33 newborn mice. All F0 mutant mice were heterozygous (*Rab44*^+/−^) and showed no apparent phenotypes. The *Rab44*^+/−^ mice were then crossed with wild-type C57BL/6 mice until the F4 generation. *Rab44*^−/−^mice (Accession No. CDB0039E: http://www2.clst.riken.jp/arg/mutant%20mice%20list.html) developed normally. For various experiments, age- and sex-matched wild-type mice were used as controls. Mice were kept under specific-pathogen-free conditions in the animal facility of Nagasaki University.

**Passive systemic anaphylaxis**

Wild type and *Rab44*^−/−^ mice were sensitized by the intraperitoneal injection of 10 µg of anti-DNP mouse monoclonal IgE (D8406, Sigma-Aldrich). After 24 h, the mice were intraperitoneally challenged with 0.8 mg of DNP-HSA (A6661, Sigma-Aldrich) and rectal temperatures were measured for 100 min using a digital thermometer (Ad1687; A&D Company Ltd). Mice were anesthetized and the venous blood samples were collected to measure plasma histamine levels using an ELISA kit (Enzo).

**Active systemic anaphylaxis**

Wild-type and *Rab44*^−/−^ mice were sensitized by the intraperitoneal injection of a mixture of 50 µg OVA (A5503, Sigma-Aldrich) and 1 mg of Alum adjuvant (A7210, Sigma-Aldrich). After 7 d, the mice were intravenously challenged with 1 mg of OVA. Rectal temperatures were then measured at 45 min after the injection using a digital thermometer.

**Degranulation assay**

BMMCs (2.5 × 10^5^ cells/well in 48-well plates) were sensitized for 5 h with 1 µg/mL anti-DNP IgE antibody and were stimulated for 20-60 min with 1-µg/mL DNP-HSA in Tyrode’s buffer (130 mM NaCl, 5 mM KCl, 1.4 mM CaCl_2_, 1 mM MgCl_2_, 5.6 mM glucose, 0.1% BSA, and 10 mM HEPES, pH 7.4). Samples were centrifuged and supernatants were collected to measure the amount of β-hexosaminidase and histamine released. To determine the total intracellular content of β-hexosaminidase, cells were lysed with 1 % Triton X-100 in Tyrode’s buffer. For degranulation assays, 50 µL of supernatant or cell lysate was incubated with 200 µL *p*-nitrophenyl-*N*-acetyl-β-D-glucosaminide (1 mM in 0.05 M sodium citrate, pH 4.5) and incubated for 60 min at 37°C. The reaction was stopped by the addition of 100 µL of 0.2 M sodium carbonate buffer, pH 10.0, and absorbance was measured at 405 nm using a microplate reader. T The percent degranulation was calculated as follows: (absorbance of culture supernatants × 100)/ (sum of absorbance of cell lysate and culture supernatant).

**Immunohistochemistry**

Immunohistochemistry were performed as described previously^9^. Briefly, five micrometer-thick sections of fixed femur were prepared from wild-type mice. Sections were blocked in PBS containing 5% normal donkey serum. Rabbit polyclonal anti-Rab44 IgG (1:1,000) was used as the primary antibody, followed by fluorescent labeling with Alexa Fluor 488-conjugated anti-rabbit IgG. Images were acquired using LSM800 confocal scanning microscope (Carl Zeiss), equipped with Airyscan and the images were processed automatically with an additional manual adjustment of plus 0.5 per channel using the Zeiss Zen Blue 2.3 software package.

**Western blot analysis**

Western blotting analysis was performed according to previously described methods^10^. Briefly, the cells were lysed in cell lysis buffer. Equal amounts of protein were subjected to SDS-PAGE followed by transfer onto a polyvinylidene difluoride membrane. The blots were blocked with 5% milk in TBS for 1 h at 25°C, incubated with primary antibodies overnight at 4°C, washed, incubated with horseradish peroxidase-conjugated secondary antibodies, and detected with Immobilon Forte Western HRP substrate (Merk-Millopore). Immunoreactive bands were analyzed using a LAS-4000 Mini imaging system (Fujifilm).

**Statistical analysis**

Quantitative data are presented as mean ± standard deviation. Statistically analyses were performed using Prism 7 (GraphPad, San Diego, CA, USA).
