# Supplementary material for: The large GTPase Rab44 regulates granule exocytosis in mast cells and IgE-mediated anaphylaxis
Source: Cell Mol Immunol. 2020 Apr 1;17(12):1287–9. doi: 10.1038/s41423-020-0413-z (PMC7784977; doi:10.1038/s41423-020-0413-z)
Supplement: Supplementary file 2 — Supplementary Table [file 41423_2020_413_MOESM2_ESM.pdf]

Supplementary Table 1. Primers in this study

Sequence Name	Sequence
**Quantitative RT-PCR**	
**mRab44-qF**	AGAGACCACACACACTCTC
**mRab44-qR**	CTCCTGTAAGTCTGTTCTTG
**mGAPDH-qF**	AACGACCCCTTCATTGACCTC
**mGAPDH-qR**	ACTGTGCCGTTGAATTTGCC
**Genotyping**	
**gtFW1**	TGCCTTACTGGAGAGAGC
**gtREV1**	CTTGAGTAGTCCTGCTGC
**gtFW2**	GCTCAGCTAAGCTGGCTC
**gtREV2**	CCTGAGGGTACGGTAAAC
